# Uncertainty Relations in the Madelung Picture Including a Dissipative Environment

**DOI:** 10.3390/e25020312

**Published:** 2023-02-08

**Authors:** Dieter Schuch, Moise Bonilla-Licea

**Affiliations:** 1 Institute of Theoretical Physics, Goethe-University Frankfurt, Max-von-Laue-Str. 1, D-60438 Frankfurt am Main, Germany; 2 CINVESTAV-IPN, Control Automático, AP 14-740, Mexico City 07000, Mexico

**Keywords:** dynamical invariant, dissipative systems, complex quantum hydrodynamics, uncertainty relations

## Abstract

In a recent paper, we have shown how in Madelung’s hydrodynamic formulation of quantum mechanics, the uncertainties are related to the phase and amplitude of the complex wave function. Now we include a dissipative environment via a nonlinear modified Schrödinger equation. The effect of the environment is described by a complex logarithmic nonlinearity that vanishes on average. Nevertheless, there are various changes in the dynamics of the uncertainties originating from the nonlinear term. Again, this is illustrated explicitly using generalized coherent states as examples. With particular focus on the quantum mechanical contribution to the energy and the uncertainty product, connections can be made with the thermodynamic properties of the environment.

## 1. Introduction

In 1926, Schrödinger published his papers on wave mechanics, introducing the equation named after him [1]. The time-dependent version [2] has the form

(1) $$i\hbar \frac{\partial }{\partial t}\psi \left(x,t\right)=\{-\frac{\hbar^{2}}{2m}\frac{\partial^{2}}{\partial x^{2}}+V\left(x,t\right)\}\psi \left(x,t\right)=H_{\mathrm{op}}\psi \left(x,t\right)$$

(our discussion will be restricted to one dimension and systems with analytic solutions, i.e., potentials *V* that are at most quadratic in the position variable), and ψ(x,t) is a complex function of space and time. Shortly after this, Madelung [3] found a reformulation in terms of two real equations that have formal similarity with equations known from hydrodynamics. For this purpose, he used the polar form

(2) $$\psi \left(x,t\right)=\sqrt{\rho (x,t)}\exp(\frac{i}{\hbar }S\left(x,t\right))$$

for the complex wave function, where the amplitude is expressed in terms of the probability density $\rho \left(x,t\right)=\psi^{*}\psi$ and the phase essentially depends on the function S(x,t) that has the dimension of action. The two equations are the continuity equation

(3) $$\frac{\partial }{\partial t}\rho +\frac{\partial }{\partial x}\left(\rho \frac{1}{m}\frac{\partial }{\partial x}S\right)=0$$

and a modified Hamilton–Jacobi equation

(4) $$\frac{\partial }{\partial t}S+\frac{1}{2m}{\left(\frac{\partial }{\partial x}S\right)}^{2}+V+V_{\mathrm{qu}}=0$$

with the so-called quantum potential

(5) $$V_{\mathrm{qu}}=-\frac{\hbar^{2}}{8m}{\left(\frac{\frac{\partial }{\partial x}\rho }{\rho }\right)}^{2}-\frac{\hbar^{2}}{4m}\frac{\partial }{\partial x}\left(\frac{\frac{\partial }{\partial x}\rho }{\rho }\right)=-\frac{\hbar^{2}}{2m}\frac{\frac{\partial^{2}}{\partial x^{2}}\sqrt{\rho }}{\sqrt{\rho }}.$$

Equations (3)–(5) are also the formal basis of Bohmian mechanics [4,5,6]; however, Bohm’s interpretation is ontologically quite different. While Madelung stuck to the conventional interpretation of quantum mechanics, Bohm introduced the concept of “hidden” variables that should convert quantum mechanics to a deterministic theory. The deterministic aspect is represented by the so-called Bohmian trajectories that are obtained via integration of the velocity field $\frac{1}{m}\frac{\partial }{\partial x}S\left(x,t\right)$ that appears in the continuity Equation (3). These trajectories are assumed to be real geometric paths of quantum particles that actually exist. However, no experiment was able to detect these trajectories. To the contrary, the experiments of last year’s Nobel laureates, those of Aspect [7,8,9] in particular, contradicted the existence of such physical trajectories. Moreover, we could show theoretically [10] that there is an ambiguity in the derivation of these trajectories that can be eliminated; but this inevitably leads to an interpretation of these trajectories in terms of descriptive statistics, thereby providing a complementary (but still probabilistic) aspect to the conventional interpretation of quantum mechanics in terms of statistical inference. Therefore, the formalism, particularly the computational aspect of Bohmian mechanics, is still very useful and has many fields of application, as shown, for example, in the interesting review article by Benseny et al. [11].

As can be seen in Equation (3), the time-evolution of the amplitude $\sqrt{\rho }$ of the wave function ψ contains its phase, S/ℏ, and Equation (4) for the phase contains the amplitude via $V_{\mathrm{qu}}$, so both equations are coupled.

In a recent paper [12], we have shown how this coupling affects the uncertainties of position and momentum and their correlation, and particularly, how the momentum uncertainty can be split into contributions from the amplitude and from the phase. In this paper, we show the influences of a dissipative environment on these properties.

Let us first specify what we mean by environment and how this environment can be taken into account in our case. In physics, one usually does not consider the whole universe, only part of it that one calls the “system.” Ideally, we assume this system to be isolated from the rest of the universe. This is the case, e.g., in classical and in quantum mechanics. However, it can also be of interest to include interactions of this system with the rest of the universe, or at least parts of it. This rest is usually called the environment, the reservoir, a heat bath or something similar. These systems, which are no longer considered isolated, are therefore also called open systems, and there are different approaches to treating such systems.

The viewpoint of there being no artificial distinction between system and environment, but of considering both together as a closed, isolated system, is closest taken by the so-called system-plus-reservoir approach. In this case, the system of interest is explicitly coupled to a (large) number of environmental degrees of freedom (often represented by harmonic oscillators). In the end, however, the environmental degrees of freedom are averaged out, and only their influence on the system is considered. This type of approach is often called the Caldeira–Leggett model [13,14] but has also been considered independently by other authors (for more details, see, e.g., [15,16]). The more environmental degrees of freedom are taken into account (in the limit infinitely many, corresponding to the so-called Markovian assumption, which actually artificially breaks the time symmetry of the system’s evolution), the better the results.

As the system-plus-reservoir approach requires extensive calculations for a large number of external degrees of freedom, possibly exceeding even the potential of powerful computers when nonlinearities are involved, and the environmental degrees of freedom are averaged out in the end anyway, a different type of approach, the effective one, takes into account only the effect of the environment on the system of interest, but not the individual interactions with the environmental degrees of freedom.

There are various approaches in this direction in the literature [17,18,19,20,21,22] using modifications of the time-dependent Schrödinger Equation (1), but most of them suffer serious shortcomings concerning their physical consequences with respect to irreversibility and correct dissipation of the system. We chose an approach that does not have these problems and start by breaking the time-reversal symmetry of the continuity Equation (3) by adding a diffusion term, thereby changing it to a Fokker–Planck-type Equation (in position space called Smoluchowski equation). With an additional separation condition, this real equation can be split into two (conjugate) complex Schrödinger-type equations with complex logarithmic nonlinearity.

The nonlinearity vanishes on average, so no imaginary contribution to the energy appears, and normalization of the corresponding wave function is still guaranteed. However, the nonlinearity has a significant influence on the analytic behavior of the time-dependence of the exact Gaussian-shaped wave-packet solutions of this nonlinear equation. Therefore, also the time-dependence of the phase and amplitude will be influenced by the additional nonlinear term.

As in our previous paper without dissipative environment [12], we again consider the uncertainties of position, momentum and their correlation; and the contributions of amplitude and phase of the wave function, but now analyze the effects of the environmental term on them.

We use these uncertainties to formulate their contributions to the energy of the system and the Heisenberg uncertainty product and their time-dependence. Using the parameters that connect the quantum system with a Brownian-motion environment, relations can also be established with thermodynamic properties of this environment.

In Section 2, our complex hydrodynamic version of Madelung’s approach, proposed in [23], will be introduced and applied in connection with the quantum uncertainties. In particular, the explicit form for generalized coherent states, i.e., the analytical Gaussian wave-packet solutions with time-dependent width will be shown.

The discussion in Section 3 focuses on the interaction with an environment. For this purpose, a time-symmetry-breaking diffusion term is used in the continuity equation, and using a particular separation condition, a nonlinear modification of the time-dependent Schrödinger equation is obtained. The properties of the complex nonlinear additional term are also pointed out.

The consequences are shown for the corresponding modified Hamilton–Jacobi equation and for the equations of motion for the wave packet maximum and width. This also elucidates the influences of real and imaginary parts of the nonlinear term on the uncertainties of position and momentum and the contributions of amplitude and phase to these quantities. This is also investigated for the Gaussian wave-packet solutions that still exist despite the logarithmic nonlinearity.

These results provide the foundation for Section 4. Based on them, the uncertainties are used to construct their contributions to the energy of the system and to the Heisenberg uncertainty product. In particular, the harmonic oscillator and the free motion, without and with environment, are compared for selected initial conditions, followed by a discussion about connections with thermodynamic properties of the environment, by way of parameters characterizing this environment.

Finally, in Section 5 the results are summarized and conclusions drawn.

## 2. Complex Madelung Picture

### 2.1. Quantum Fluctuations in the Madelung Picture

In [23], it was shown how Madelung’s hydrodynamic version of quantum mechanics can be formulated in terms of complex quantities that are obtained by applying a quantum mechanical operator $F_{\mathrm{op}}$ onto a quantum state or wave function 〈a|ψ(x,t)〉 in the {|a〉} representation (in this paper “*a*” is position “*x*” and 〈x|ψ(x,t)〉=ψ(x,t)) and dividing the result by 〈a|ψ(x,t)〉—in our case,

(6) $$F=\frac{F_{\mathrm{op}}\psi \left(x,t\right)}{\psi (x,t)}=F_{R}+iF_{I}.$$

The result is, in general, complex; however, the mean value of the imaginary part always vanishes, meaning $\langle F_{I}\rangle =0$; i.e., it cannot be observed directly. Nevertheless, e.g., $\langle F_{I}^{2}\rangle \neq 0$ or $\langle \frac{\partial }{\partial x}F_{I}\rangle \neq 0$ is possible; see Equation (32) (for further details see [12,23,24]).

In position space, the relevant operators and corresponding complex quantities are

(7) $$\begin{array}{ccc} X_{\mathrm{op}}=x\in R & ⟶ & X=x\in R \end{array}$$

(8) $$\begin{array}{ccc} P_{\mathrm{op}}=-i\hbar \frac{\partial }{\partial x} & ⟶ & P=\frac{\partial }{\partial x}S-i\frac{\hbar }{2}\frac{\frac{\partial }{\partial x}\rho }{\rho }=P_{R}+iP_{I} \end{array}$$

(9) $$\begin{array}{ccc} V_{\mathrm{op}}=\frac{m}{2}\omega_{0}^{2}X_{\mathrm{op}}^{2} & ⟶ & V=\frac{m}{2}\omega_{0}^{2}x^{2} \end{array}$$

(10) $$\begin{array}{ccc} T_{\mathrm{op}}=-\frac{\hbar^{2}}{2m}\frac{\partial^{2}}{\partial x^{2}} & ⟶ & T=\frac{1}{2m}\left(P^{2}\right)=\frac{1}{2m}\left[{\left(P\right)}^{2}-\frac{\hbar }{i}\frac{\partial }{\partial x}P\right] \end{array}$$

(11) $$\begin{array}{ccc} E_{\mathrm{op}}=i\hbar \frac{\partial }{\partial t} & ⟶ & E=-\frac{\partial }{\partial t}S+i\frac{\hbar }{2}\frac{\frac{\partial }{\partial t}\rho }{\rho }. \end{array}$$

The difference between the quantity corresponding to the square of an operator and the square of the quantity corresponding to this operator does not vanish, $\left(F^{2}\right)\neq {\left(F\right)}^{2}$, as can be seen in (10) for the momentum operator in the expression for the kinetic energy.

This difference originates from the non-locality of quantum mechanics, which was already pointed out in [12].

In order to compute the uncertainty of an observable, expressed in a given representation by an operator $F_{\mathrm{op}}$, one must calculate

(12) $$\sigma_{F}^{2}=\langle F_{\mathrm{op}}^{2}\rangle -{\langle F_{\mathrm{op}}\rangle }^{2}=\int_{-\infty }^{+\infty }dx\;\psi^{*}F_{\mathrm{op}}^{2}\psi -{\left[\int_{-\infty }^{+\infty }dx\;\psi^{*}F_{\mathrm{op}}\psi \right]}^{2}$$

(here for position representation), where pointed brackets denote mean values. This can be expressed in terms of our Madelung quantities as

(13) $$\sigma_{F}^{2}=\int_{-\infty }^{+\infty }dx\;\rho \left(x,t\right)\left(F^{2}\right)-{\left[\int_{-\infty }^{+\infty }dx\;\rho F\right]}^{2}.$$

As mentioned above and in [12], the mean values of the imaginary parts always vanish, $\langle F_{I}\rangle =0$, so only the real parts of the Madelung quantities are to be considered for the uncertainties.

In the case of the momentum uncertainty, the real part of $\left(P^{2}\right)$ can be expressed via the real part of the kinetic energy as

(14) $$\sigma_{p}^{2}=2m\int_{-\infty }^{+\infty }dx\;\rho \left(x,t\right)T_{R}-{\langle p\rangle }^{2}$$

with

(15) $$T_{R}=\frac{1}{2m}\left(P_{R}^{2}-P_{I}^{2}+\hbar \frac{\partial }{\partial x}P_{I}\right)=\frac{1}{2m}P_{R}^{2}+V_{\mathrm{qu}},$$

leading to

(16) $$\sigma_{p}^{2}=\left[\int_{-\infty }^{+\infty }dx\;\rho \left(x,t\right)P_{R}^{2}-{\langle p\rangle }^{2}\right]+2m\int_{-\infty }^{+\infty }dx\;\rho \left(x,t\right)V_{\mathrm{qu}}=\sigma_{p,ph}^{2}+\sigma_{p,am}^{2},$$

i.e., one contribution from the phase and one from the amplitude.

The contribution from the phase depends on $P_{R}=\frac{\partial }{\partial x}S$ and vanishes if *S* does not explicitly depend on position; the contribution from the amplitude is always present (in general, plane wave-like functions are an exception).

Since the position operator is only a c-number, not a differential operator, there is no contribution from the phase to $\sigma_{x}^{2}$.

Finally, the correlation of position and momentum uncertainties, $\sigma_{xp}$, is given in the Madelung picture. From

(17) $$\sigma_{xp}=\frac{1}{2}\langle X_{\mathrm{op}}P_{\mathrm{op}}-P_{\mathrm{op}}X_{\mathrm{op}}\rangle -\langle x\rangle \langle p\rangle =\langle X_{\mathrm{op}}P_{\mathrm{op}}-i\frac{\hbar }{2}\rangle -\langle x\rangle \langle p\rangle$$

follows with definitions (7) and (8)

(18) $$\sigma_{xp}=\int_{-\infty }^{+\infty }dx\;\rho \left(x,t\right)xP-\langle x\rangle \langle p\rangle -i\frac{\hbar }{2}.$$

Taking into account the form of $P_{I}$, as given in (8), and via integration by parts, it can be shown that for square integrable wave functions,

(19) $$\int_{-\infty }^{+\infty }dx\;\rho \left(x,t\right)xP_{I}=\frac{\hbar }{2}$$

is fulfilled, guaranteeing that expression (18) remains real. The correlation of position and momentum uncertainties can then be written in the form

(20) $$\sigma_{xp}=\int_{-\infty }^{+\infty }dx\;\rho \left(x,t\right)xP_{R}-\langle x\rangle \langle p\rangle =\int_{-\infty }^{+\infty }dx\rho \left(x,t\right)\left(x-\langle x\rangle \right)\left(P_{R}-\langle p\rangle \right).$$

It is important to note that although $\langle P_{R}\rangle =\langle \frac{\partial }{\partial x}S\rangle =\langle p\rangle$ is valid, the integral on the very rhs of (20) does not necessarily vanish if the phase S/ℏ explicitly depends on the position variable in a nonlinear way (as in the case of generalized coherent states, to be discussed subsequently). Therefore, the correlation depends entirely on the phase of the wave function and not at all on the amplitude.

For a more detailed illustration of the uncertainties and their correlations, we now consider exact analytic solutions of the time-dependent Schrödinger equation, so-called generalized coherent states, i.e., Gaussian shaped wave packets with time-dependent width.

### 2.2. Uncertainties and Their Correlations for Generalized Coherent States

For potentials that are at most quadratic in position variable (such as our potential *V* in (9)), Gaussian wave packets with time-dependent maximum and width, also called generalized coherent states, are exact analytic solutions of the time-dependent Schrödinger equation. They can be written in the form

(21) $$\begin{array}{ccc} \psi (x,t) & = & N\left(t\right)\exp\left[\frac{i}{\hbar }\left(\frac{m}{2}C{\tilde{x}}^{2}+\langle p\rangle \tilde{x}+K\left(t\right)\right)\right]\\ & = & N\left(t\right)\exp\left[-\frac{{\tilde{x}}^{2}}{\sigma_{x}^{2}}+\frac{i}{\hbar }\left(\frac{m}{2}C_{R}{\tilde{x}}^{2}+\langle p\rangle \tilde{x}+K\left(t\right)\right)\right] \end{array}$$

with $\tilde{x}=x-\langle x\rangle =x-\eta \left(t\right)$ and the complex time-dependent coefficient $C\left(t\right)=C_{R}+iC_{I}$ of the quadratic term in the exponent, which has the dimension of inverse time, i.e., frequency, such as $\omega_{0}$ (see Equation (23)). N(t) is a time-dependent normalization factor, not relevant for the following; this is also true for the purely time-dependent function K(t).

The maximum of the Gaussian function, located at 〈x〉=η(t), follows the classical Newtonian equation of motion:

(22) $$\ddot{\eta }+\omega_{0}^{2}\eta =0$$

the complex function C(t) fulfills the nonlinear Riccati equation

(23) $$\dot{C}+C^{2}+\omega_{0}^{2}=0,$$

where overdots denote time-derivatives.

The imaginary part of C is related with the position uncertainty via

(24) $$C_{I}=\frac{\hbar }{2m}\frac{1}{\sigma_{x}^{2}}=\frac{1}{\alpha^{2}\left(t\right)}$$

with $\sigma_{x}^{2}=\langle x^{2}\rangle -{\langle x\rangle }^{2}=\langle {\tilde{x}}^{2}\rangle$ and a new variable α(t) that is proportional to the wave packet width and will be useful in our later discussion.

From the imaginary part of the Riccati equation,

(25) $${\dot{C}}_{I}+2C_{R}C_{I}=0,$$

follows

(26) $$C_{R}=-\frac{1}{2}\frac{\frac{d}{dt}C_{I}}{C_{I}}=\frac{1}{2}\frac{\frac{d}{dt}\sigma_{x}^{2}}{\sigma_{x}^{2}}=\frac{\dot{\alpha }}{\alpha }.$$

The real part of the Riccati equation can then be written in terms of α(t) as a so-called real nonlinear Ermakov equation:

(27) $$\ddot{\alpha }+\omega_{0}^{2}\alpha^{2}=\frac{1}{\alpha^{3}},$$

that determines the time-evolution of the wave packet width.

The density ρ(x,t) corresponding to the wave packet (21) can be written as

(28) $$\rho \left(x,t\right)={\left|N\left(t\right)\right|}^{2}\exp\left[-\frac{m}{\hbar }C_{I}{\tilde{x}}^{2}\right]=\sqrt{\frac{1}{2\pi \sigma_{x}^{2}}}\exp\left[-\frac{{\tilde{x}}^{2}}{\sigma_{x}^{2}}\right].$$

Therefore, the position uncertainty is fixed by the width of the density ρ(x,t). This width, however, can be time-dependent; and the time-dependence enters the coefficient $C_{R}\left(t\right)$ in the phase of the wave function, thereby contributing to the momentum uncertainty. Using our complex Madelung picture, this can be expressed as

(29) $$\sigma_{p}^{2}=\langle {\left(P^{2}\right)}_{R}\rangle -{\langle p\rangle }^{2}=\langle P_{R}^{2}-P_{I}^{2}+\hbar \frac{\partial }{\partial x}P_{I}\rangle -{\langle p\rangle }^{2}$$

with

(30) $$P_{R}=\frac{\partial }{\partial x}S=mC_{R}\tilde{x}+\langle p\rangle$$

and

(31) $$P_{I}=-\hbar \frac{\frac{\partial }{\partial x}\rho }{\rho }=mC_{I}\tilde{x}=\frac{\hbar }{2}\frac{\tilde{x}}{\sigma_{x}^{2}}.$$

As shown above, the momentum uncertainty has two contributions, one from the amplitude and one from the phase. In our case, these now have the explicit form

(32) $$\sigma_{p,am}^{2}=\langle P_{I}^{2}\rangle -\langle \hbar \frac{\partial }{\partial x}P_{I}\rangle =\frac{\hbar^{2}}{4}\frac{1}{\sigma_{x}^{2}}=\frac{m\hbar }{2}\frac{1}{\alpha^{2}}$$

and

(33) $$\sigma_{p,ph}^{2}=\langle P_{R}^{2}\rangle -{\langle P_{R}\rangle }^{2}=\frac{\sigma_{xp}^{2}}{\sigma_{x}^{2}}=m\frac{\hbar }{2}{\dot{\alpha }}^{2}.$$

For the last expression on the rhs of (33), it has been taken, according to (20), that the position-momentum correlation, $\sigma_{xp}$ can be written as

(34) $$\sigma_{xp}=\langle \tilde{x}P_{R}\rangle =mC_{R}\langle {\tilde{x}}^{2}\rangle =m\frac{d}{dt}\sigma_{x}^{2}=\frac{\hbar }{2}\dot{\alpha }\alpha ,$$

from which it follows that $C_{R}$ can be expressed as

(35) $$C_{R}=\frac{1}{m}\frac{\sigma_{xp}}{\sigma_{x}^{2}}=\frac{\dot{\alpha }}{\alpha }.$$

Therefore, the uncertainty product can be written as

(36) $$U_{L}=\sigma_{x}^{2}\sigma_{p}^{2}=\sigma_{x}^{2}(\sigma_{p,am}^{2}+\sigma_{p,ph}^{2})=\frac{\hbar^{2}}{4}[1+({\frac{2}{\hbar }\sigma_{xp})}^{2})]=\frac{\hbar^{2}}{4}[1+{\left(\dot{\alpha }\alpha \right)}^{2}];$$

i.e., the minimum uncertainty $\frac{\hbar^{2}}{4}$ only depends on the amplitude of the wave function and is hence also guaranteed for time-independent width. If the uncertainty product is larger, this additional contribution originates entirely from the phase and is, in the case of our generalized coherent states, related to the time-dependence of the wave packet width that, again, reflects the correlation of position and momentum.

Thus, the uncertainties that determine the evolution of the wave packet are essentially the position uncertainty $\sigma_{x}^{2}$, which guarantees the minimum uncertainty product, and the position momentum correlation $\sigma_{xp}$, which provides additional contributions that essentially depend on the phase of the wave packet. If at the initial time t=0, the initial value of $\dot{\alpha }$ vanishes, ${\dot{\alpha }}_{0}=0$, which is usually the case for the harmonic oscillator and the free motion, the contribution from $\sigma_{xp}$ to the uncertainty product also vanishes, $U_{L}\left(0\right)=\frac{\hbar^{2}}{4}$, and the wave packet is initially a so-called minimum uncertainty coherent state (MUCS).

The complex quantity C(t) that determines the time-evolution of the uncertainties via a complex Riccati equation can then be written using $\sigma_{x}^{2}$, $\sigma_{xp}$ and α and $\dot{\alpha }$ as

(37) $$C=\frac{1}{m}\frac{\sigma_{xp}}{\sigma_{x}^{2}}+i\frac{\hbar }{2m}\frac{1}{\sigma_{x}^{2}}=\frac{\dot{\alpha }}{\alpha }+i\frac{1}{\alpha^{2}}.$$

## 3. Open Systems with Irreversibility and Dissipation

There are different approaches to treating open systems classically and quantum mechanically (for a survey, see, e.g., [16]). One possibility is to couple the system of interest to a bath of environmental degrees of freedom, e.g., harmonic oscillators where the number of these degrees of freedom must be very large in order to obtain physically sound results, and in the end, they are averaged out. This leads to extensive and costly calculations that can even go beyond the capacities of current computers when nonlinearities are involved.

A different viewpoint is taken by so-called effective approaches where the individual degrees of freedom of the environment are not taken into account explicitly, only their effect on the system of interest. Amongst those are attempts to modify the classical Lagrange–Hamilton formalism in a way that additional dissipative friction forces, usually linear proportional to velocity or momentum, can be incorporated into the equation of motion of the system. In general, this involves non-canonical transformations with corresponding challenges [16,17,18]. A quantum mechanical version is then obtained via canonical quantization, leading to modified, often explicitly time-dependent but usually linear, modifications of the Schrödinger equation.

A different class of effective approaches circumvents the problems with non-canonical aspects and starts already on the quantum mechanical level by adding terms to the Hamiltonian operator that yield the above-mentioned friction forces, leading to nonlinear Schrödinger equations. One way of obtaining the additional terms is to require that these provide, according to Ehrenfest, the desired friction force in the equation of motion. In our Madelung picture, the equation that leads to the force is the modified Hamilton–Jacobi Equation (3). Taking the gradient of this equation and using $\frac{\partial }{\partial x}S=P_{R}$ provides a kind of Euler equation with substantial time-derivative $\frac{D}{Dt}$ in a co-moving frame:

(38) $$\left(\frac{\partial }{\partial t}+\frac{1}{m}P_{R}\frac{\partial }{\partial x}\right)P_{R}=\frac{D}{Dt}P_{R}=-\frac{\partial }{\partial x}\left(V+V_{\mathrm{qu}}\right).$$

A friction force should appear as an additional term proportional to $P_{R}$ (with negative sign) on the rhs of Equation (38). As this requirement leaves room for ambiguity, there are several approaches in the literature to achieve this goal [19,20,21,22], but most of them suffer serious shortcomings and produce unphysical results.

Another problematic aspect is the fact that (38) is related only to the real part of the Schrödinger equation, so the additional friction force in (38) should originate from an additional real term in the modified Schrödinger equation. However, since real terms do not affect the Madelung equation representing the imaginary part of the Schrödinger equation, this would still remain a reversible continuity equation, contradicting the observation that dynamics of open system is irreversible, particularly if friction is involved.

Therefore, we use a different approach, starting in the Madelung picture with the continuity Equation (2) and breaking the time-reversal symmetry by introducing an additional diffusion term:

(39) $$\begin{array}{ccc} \frac{\partial }{\partial t}\rho +\frac{\partial }{\partial x}\left(\rho \frac{1}{m}\frac{\partial }{\partial x}S\right)-D\frac{\partial^{2}}{\partial x^{2}}\rho & & \\ =\frac{\partial }{\partial t}\rho +\frac{\partial }{\partial x}\left[\rho \left(\frac{1}{m}\frac{\partial }{\partial x}S-D\frac{\frac{\partial }{\partial x}\rho }{\rho }\right)\right] & = & 0. \end{array}$$

This turns the reversible continuity equation into an irreversible Fokker–Planck-type equation in position space called the Smoluchowski equation, which is known from the description of Brownian motion. An equivalent description of Brownian motion in the trajectory picture via the Langevin equation involves the above-mentioned friction force that will also be compatible with our approach, as will be shown below.

The convection velocity field $\frac{1}{m}\frac{\partial }{\partial x}S=\frac{1}{m}P_{R}$ of (2) is now replaced by the total velocity field

(40) $$\upsilon_{T}=\frac{1}{m}\frac{\partial }{\partial x}S-D\frac{\frac{\partial }{\partial x}\rho }{\rho }=\upsilon_{\mathrm{con}}+\upsilon_{\mathrm{diff}}$$

with the additional diffusion velocity $-D\frac{\frac{\partial }{\partial x}\rho }{\rho }$, where *D* is the diffusion coefficient characterizing the environment.

In conventional quantum mechanics, the continuity equation for $\rho =\psi^{*}\psi$ is obtained by combining the Schrödinger equation for ψ with its complex conjugate for $\psi^{*}$. Thus, the question arises of whether this procedure can also be reversed to obtain the complex modified Schrödinger equation corresponding to (39) by separation of this equation. A way to achieve this without the diffusion term was shown by Madelung [25] and Mrowka [26]. Unfortunately, due to the diffusion term, ψ and $\psi^{*}$ are coupled, and therefore, the above-mentioned method cannot be applied in general. However, there might be special conditions that can be fulfilled by the diffusion term to still allow separation.

One such separation condition is given by

(41) $$-D\frac{\frac{\partial^{2}}{\partial x^{2}}\rho }{\rho }=\gamma \left(\ln\rho -\langle \ln\rho \rangle \right)$$

that is also fulfilled particularly for Gaussian functions—those we consider in our analysis. The subtraction of the mean value of lnρ is necessary to guarantee normalizability of the solutions of the corresponding Schrödinger equation, as the diffusion term leads to an imaginary contribution in the Hamiltonian, thereby turning it into a non-Hermitian one.

Separation of (39), using (41), leads to a nonlinear Schrödinger equation with complex logarithmic nonlinearity:

(42) $$\begin{array}{ccc} i\hbar \frac{\partial }{\partial t}\psi_{\mathrm{NL}}\left(x,t\right) & = & \left\{-\frac{\hbar^{2}}{2m}\frac{\partial^{2}}{\partial x^{2}}+V+\gamma \frac{\hbar }{i}\left(\ln\psi -\langle \ln\psi \rangle \right)\right\}\psi_{\mathrm{NL}}\left(x,t\right)\\ & = & H_{\mathrm{NL},\mathrm{op}}\psi \left(x,t\right)=\left(H_{L,\mathrm{op}}+W\right)\psi \left(x,t\right) \end{array}$$

with

(43) $$W=\gamma \frac{\hbar }{i}\left(\ln\psi -\langle \ln\psi \rangle \right)=\frac{\gamma }{2}\frac{\hbar }{i}\left(\ln\frac{\psi }{\psi^{*}}-\langle \ln\frac{\psi }{\psi^{*}}\rangle \right)-i\frac{\gamma }{2}\hbar \left(\ln\rho -\langle \ln\rho \rangle \right)=W_{R}+iW_{I}.$$

Due to the appearance of the imaginary part $iW_{I}$, the nonlinear Hamiltonian is non-Hermitian. This usually leads to problems with the normalizability of the corresponding wave functions. In a similar approach to describe open dissipative systems, Gisin [27,28], via a Master equation for the wave function, also arrived at an imaginary term in the Hamiltonian, but enforced normalizability via subtraction of its mean value. In our case, this mean value consistently originates from the separation condition (41). Gisin’s approach contains some ambiguities; and also his expression for the decay of the system’s energy disagrees with the one known, but it was a step in the right direction.

A nonlinear Schrödinger equation, containing only our imaginary contribution without the real (dissipative) term, was obtained independently by Beretta [29,30,31] (but in the context of density operators) with the aim of describing non-equilibrium systems (without dissipation).

In an attempt to reduce the treatment of the quantum mechanical many-body wave function to a discussion in terms of a single-particle wave function, Oriols [32] also arrived at an imaginary term in the corresponding Schrödinger equation. However, as admitted in [11] concerning this imaginary term, the “numerical values are in principle unknown and need some educated guesses” [32,33].

Further details concerning the derivation of Equation (42), its properties and connections with similar nonlinear approaches can be found in [16].

As a result of the subtraction of the mean value of lnψ, the mean value of the nonlinear term vanishes; therefore, the mean value of energy is still the mean value of the operators of kinetic and potential energies. However, the mean values are now calculated with the solution $\psi_{\mathrm{NL}}\left(x,t\right)$ of the nonlinear Schrödinger equation (Equation (42)). The nonlinear term obviously influences the dynamics of phase and amplitude, and thus of the maximum and width of the wave packet. The details will be obvious when the equations of motion of these quantities are examined.

The additional nonlinear term also has a real contribution that is not arbitrary but fixed by the separation condition and has a clear physical meaning. A first idea of this meaning can be obtained by looking at the second of Madelung’s equations, the modified Hamilton–Jacobi equation. Due to the real part of the nonlinear term, it turns into

(44) $$\frac{\partial }{\partial t}S+\frac{1}{2m}{\left(\frac{\partial }{\partial x}S\right)}^{2}+V+V_{\mathrm{qu}}+\gamma \left(S-\langle S\rangle \right)=0.$$

Taking the gradient of this equation now yields the Euler Equation (38) with an additional friction term that is linearly proportional to momentum, as known from the Langevin equation, but without putting it in by the assumption of a corresponding “friction potential”, like in similar approaches [19,20,21,22] mentioned above:

(45) $$\left(\frac{\partial }{\partial t}+\frac{1}{m}P_{R}\frac{\partial }{\partial x}\right)P_{R}=\frac{D}{Dt}P_{R}=-\frac{\partial }{\partial x}\left(V+V_{\mathrm{qu}}\right)-\gamma P_{R}.$$

Considering now the Gaussian solutions of the nonlinear Schrödinger Equation (42) in the form (21), the additional complex nonlinear term *W* can be expressed as

(46) $$\begin{array}{ccc} W & = & \gamma \frac{\hbar }{i}\left(\ln\psi_{\mathrm{NL}}-\langle \ln\psi_{\mathrm{NL}}\rangle \right)=\gamma \left\{\frac{m}{2}\left[C_{R}\left({\tilde{x}}^{2}-\langle {\tilde{x}}^{2}\rangle \right)\right]+\langle p\rangle \tilde{x}\right\}\\ & + & i\gamma \frac{m}{2}C_{I}\left({\tilde{x}}^{2}-\langle {\tilde{x}}^{2}\rangle \right)=W_{R}+iW_{I} \end{array}$$

where the negative gradient (in one dimension) yields

(47) $$-\frac{\partial }{\partial x}W=-\gamma \left\{mC_{R}\tilde{x}+\langle p\rangle \right\}-i\gamma mC_{I}\tilde{x},$$

showing that its mean value is real and provides the friction force in the Ehrenfest equation; i.e.,

(48) $$\langle -\frac{\partial }{\partial x}W\rangle =-\gamma \langle p\rangle .$$

After inserting the Gaussian ansatz (21) into the nonlinear Schrödinger Equation (42), the corresponding equations of motion for maximum and width of the wave packet can be obtained as

(49) $$\begin{array}{ccc} \ddot{\eta }+\gamma \dot{\eta }+\omega_{0}^{2}\eta & = & 0 \end{array}$$

(50) $$\begin{array}{ccc} \dot{C}+\gamma C+C^{2}+\omega_{0}^{2} & = & 0. \end{array}$$

The friction force in (49) was already mentioned. Important for the uncertainties, however, is the Riccati Equation (50) with the additional linear term γC. Splitting (50) into real and imaginary parts from the imaginary part follows:

(51) $${\dot{C}}_{I}+2C_{R}C_{I}+\gamma C_{I}=0,$$

or

(52) $$C_{R}=-\frac{1}{2}\frac{{\dot{C}}_{I}}{C_{I}}-\frac{\gamma }{2}=\frac{\dot{\alpha }}{\alpha }-\frac{\gamma }{2},$$

where the definition $C_{I}=\frac{1}{\alpha^{2}}$ has been unchanged, as in the case without environment. This shows that $C_{R}$, which is crucial for the contribution of the **phase** to the uncertainties, is changed by $\gamma C_{I}$, which originates from the imaginary part of *W*, and thus from the Smoluchowski equation for the **amplitude**!

On the other hand, the real part of (50) leads to

(53) $${\dot{C}}_{R}+\gamma C_{R}+C_{R}^{2}-C_{I}^{2}+\omega_{0}^{2}=0$$

which can be written with $C_{I}$ and $C_{R}$ expressed in terms of α as a modified Ermakov equation:

(54) $$\ddot{\alpha }+\left(\omega_{0}^{2}-\frac{\gamma^{2}}{4}\right)\alpha =\frac{1}{\alpha^{3}},$$

that is identical to (27); only $\omega_{0}^{2}$ is replaced by the correct reduced frequency $\Omega^{2}=\left(\omega_{0}^{2}-\frac{\gamma^{2}}{4}\right)$ of the damped harmonic oscillator.

Here, it is exactly the other way round; the equation for α, and thus the wave packet width that characterizes the **amplitude**, is influenced by $\gamma C_{R}$, which originates from the real part of *W*, the one that corresponds to the modified Hamilton–Jacobi equation for the **phase**.

Thus, there is an **additional coupling** of phase and amplitude due to interaction with the environment.

## 4. Consequences for Energy and Uncertainty Product

### 4.1. Energy Contributions from Uncertainties

The calculation of the energy as mean value of the Hamiltonian, also for the open system, is reduced to the calculation of the mean values of kinetic and potential energies, as 〈W〉=0; i.e.,

(55) $$\begin{array}{ccc} E & = & \langle T\rangle +\langle V\rangle =\frac{1}{2m}\langle p^{2}\rangle +\frac{m}{2}\omega_{0}^{2}\langle x^{2}\rangle \\ & = & \frac{1}{2m}{\langle p\rangle }^{2}+\frac{m}{2}\omega_{0}^{2}{\langle x\rangle }^{2}+\frac{1}{2m}\langle {\tilde{p}}^{2}\rangle +\frac{m}{2}\omega_{0}^{2}\langle {\tilde{x}}^{2}\rangle =E_{\mathrm{cl}}+\tilde{E}. \end{array}$$

The classical contribution is determined by the mean values of position and momentum, and thus by the solution of Equation (49) for η(t)=〈x〉. The contribution from the uncertainties depends on $\langle {\tilde{p}}^{2}\rangle =\sigma_{p}^{2}$ and $\langle {\tilde{x}}^{2}\rangle =\sigma_{x}^{2}$, which can be obtained by solving the complex Riccati Equation (50). To consider the complete general solution of this equation for all possible initial conditions would be much too complex and beyond the scope of this paper (further details can be found in Appendix B of [16]). In the following, characteristic examples shall be discussed, which show the influences of the environment on the dynamics of the quantities that depend on the uncertainties.

First, the contribution to the energy of the harmonic oscillator is determined without and with environmental effects, and the results are compared. In both cases, the energy depends on the uncertainties via

(56) $$\tilde{E}=\frac{1}{2m}\sigma_{p}^{2}+\frac{m}{2}\omega_{0}^{2}\sigma_{x}^{2}.$$

Without environment, the Riccati equation (Equation (23)), obtained from the linear Schrödinger equation, must be solved. A particular solution, $\tilde{C}$ can be found easily, assuming it to be constant, leading to

(57) $$\tilde{C}=\pm i\omega_{0}=i{\tilde{C}}_{I}=i\frac{1}{\alpha_{0}^{2}},$$

where only the plus-sign is physically relevant, as the minus-sign would cause divergence of the wave packet; the real part vanishes: ${\tilde{C}}_{R}=0$. Consequently, the uncertainties take the form:

(58) $$\begin{array}{ccc} \sigma_{p,L}^{2} & = & \frac{m\hbar }{2}\left[{\dot{\alpha }}^{2}+\frac{1}{\alpha^{2}}\right]=\frac{m\hbar }{2}\frac{1}{\alpha_{0}^{2}}=\frac{m}{2}\hbar \omega_{0}, \end{array}$$

(59) $$\begin{array}{ccc} \sigma_{x,L}^{2} & = & \frac{\hbar }{2m}\alpha_{0}^{2}=\frac{\hbar }{2m\omega_{0}}, \end{array}$$

where the subscript L refers to the linear Schrödinger equation providing, via (56), simply the energy of the ground state:

(60) $${\tilde{E}}_{L}=\frac{\hbar }{2}\omega_{0}.$$

The general solution of Riccati Equation (23) can be found via $C\left(t\right)=\tilde{C}+V\left(t\right)$, where the complex time-dependent function V(t) fulfills the homogeneous nonlinear Bernoulli equation:

(61) $$\dot{V}+2\tilde{C}V+V^{2}=0.$$

For the following discussion, however, the particular solution $\tilde{C}$ is sufficient. The general solution is discussed in [16].

Including the dissipative environment, the Riccati equation (Equation (50)) must be solved. Considering, again, a constant particular solution $\tilde{C}$, one obtains

(62) $$\tilde{C}=-\frac{\gamma }{2}\pm i\Omega$$

where for the negative imaginary part, the same applies as in the previous case, and $\Omega =\sqrt{\omega_{0}^{2}-\frac{\gamma^{2}}{4}}$ (we restrict our discussion to undercritical damping, i.e., $\omega_{0}>\frac{\gamma }{2}$).

With $C_{R}=-\frac{\gamma }{2}$ and $C_{I}=\Omega =\frac{1}{\alpha_{0}^{2}}$, the uncertainties corresponding to the nonlinear Schrödinger equation are

(63) $$\begin{array}{ccc} \sigma_{p,\mathrm{NL}}^{2} & = & \frac{m\hbar }{2}\left[\left({\dot{\alpha }}^{2}-\frac{\gamma }{2}\alpha \right)+\frac{1}{\alpha^{2}}\right]=\frac{m\hbar }{2}\left[\frac{\gamma^{2}}{4}\alpha_{0}^{2}+\frac{1}{\alpha_{0}^{2}}\right]=\frac{m\hbar }{2}\frac{\omega_{0}}{\Omega } \end{array}$$

(64) $$\begin{array}{ccc} \sigma_{x,\mathrm{NL}}^{2} & = & \frac{\hbar }{2m}\alpha_{0}^{2}=\frac{\hbar }{2m\Omega }, \end{array}$$

leading to

(65) $${\tilde{E}}_{\mathrm{NL}}=\frac{\hbar }{2}\frac{\omega_{0}^{2}}{\Omega }=\frac{\hbar }{2}\omega_{0}\left(\frac{\omega_{0}}{\Omega }\right)>\frac{\hbar }{2}\omega_{0}={\tilde{E}}_{L},$$

i.e., a ground state energy that is larger than without environment. That resembles the situation in the case of Brownian motion where the final energy after dissipation of the center of mass energy of the Brownian particle is not zero, but $\frac{1}{2}kT$ due to the back-transfer of energy from the environment at temperature *T* caused by a stochastic fluctuating force (*k* means Boltzmann’s constant). The mean value of this force vanishes, but not its contribution to the energy, which depends on the mean value of the square of this force.

It will now be shown how the excess energy in (65) relates to the properties of the environment. For this purpose, ${\tilde{E}}_{\mathrm{NL}}$ is rewritten in the form

(66) $${\tilde{E}}_{\mathrm{NL}}=\frac{\hbar }{2}\Omega \left(1+\frac{\frac{\gamma^{2}}{4}}{\Omega^{2}}\right)=\frac{\hbar }{2}\Omega +m\frac{\gamma^{2}}{4}\sigma_{x}^{2}.$$

By inserting our Gaussian wave packet into the separation condition (41) for ρ, one obtains the diffusion coefficient *D* as

(67) $$D=\frac{\gamma }{2}\sigma_{x}^{2}.$$

On the other hand, *D* is characteristic for the properties of the environment. Assuming a Brownian motion situation, the diffusion coefficient is given by the Einstein relation:

(68) $$D=\frac{kT}{m\gamma }.$$

If we consider the environment that interacts with our quantum system to be such a Brownian one, which is reasonable, since the friction force we obtain is the same as in the Langevin equation, then *D* in (67) can be set equal to *D* in (68). Consequently, the excess energy in (66) can be written as

(69) $$m\frac{\gamma^{2}}{4}\sigma_{x}^{2}=m\frac{\gamma }{2}D=m\frac{\gamma }{2}\frac{kT}{m\gamma }=\frac{1}{2}kT,$$

which is in agreement with the aforementioned energy back-transfer from the environment due to the stochastic fluctuations. In our case, this energy contribution is connected with the diffusion coefficient in the Smoluchowski equation for ρ(t), where ρ also represents a statistical aspect of the system similar to the properties of the stochastic force.

Next, the situation for the free motion, i.e., V=0, shall be considered. For the linear Schrödinger equation without environmental effects, there is no constant particular solution $\tilde{C}$, but also for time-dependent C(t), the corresponding Riccati equation can be solved easily, leading to

(70) $$\begin{array}{ccc} \sigma_{x,L}^{2} & = & \sigma_{x,0}^{2}\left[1+{\left(\beta_{0}t\right)}^{2}\right], \end{array}$$

(71) $$\begin{array}{ccc} \sigma_{p,L}^{2} & = & \sigma_{p,0}^{2}=\frac{m\hbar }{2}\beta_{0}=\mathrm{const}. \end{array}$$

and

(72) $${\tilde{E}}_{L}=\frac{\hbar }{4}\beta_{0},$$

where $\beta_{0}=\frac{1}{\alpha_{0}^{2}}=\frac{\hbar }{2m\sigma_{x,0}^{2}}$ has the dimension of a frequency and $\sigma_{x,0}^{2}$ is the constant initial position uncertainty.

Including the environmental effect, there are now, also for V=0, constant particular solutions for the Riccati Equation (50) corresponding to the nonlinear Schrödinger equation:

(73) $$\tilde{C}=-\frac{\gamma }{2}\pm \frac{\gamma }{2},$$

with ${\tilde{C}}_{+}=0$ and ${\tilde{C}}_{-}=-\gamma$, whereas for γ=0, only $\tilde{C}=0$ exists; i.e., the environment causes a kind of bifurcation.

For the uncertainties and energy contributions, this means that there are now two different solutions.

For ${\tilde{C}}_{+}=0$, one obtains (for details see [16]):

(74) $$\begin{array}{ccc} \sigma_{x,+}^{2} & = & \sigma_{x,0}^{2}\left[e^{\gamma t}+{\left(\frac{\beta_{0}}{\gamma /2}\right)}^{2}\sinh^{2}\frac{\gamma }{2}t\right], \end{array}$$

(75) $$\begin{array}{ccc} \sigma_{p,+}^{2} & = & \frac{m\hbar }{2}\beta_{0}e^{-\gamma t} \end{array}$$

and

(76) $${\tilde{E}}_{+}=\frac{\hbar }{4}\beta_{0}e^{-\gamma t},$$

for ${\tilde{C}}_{-}=-\gamma$, these quantities are

(77) $$\begin{array}{ccc} \sigma_{x,-}^{2} & = & \sigma_{x,0}^{2}\left[e^{-\gamma t}+{\left(\frac{\beta_{0}}{\gamma /2}\right)}^{2}\sinh^{2}\frac{\gamma }{2}t\right], \end{array}$$

(78) $$\begin{array}{ccc} \sigma_{p,-}^{2} & = & \frac{m\hbar }{2}\beta_{0}\left[1+{\left(\frac{\gamma }{\beta_{0}}\right)}^{2}\right]e^{-\gamma t} \end{array}$$

and

(79) $${\tilde{E}}_{-}=\frac{\hbar }{4}\beta_{0}\left[1+{\left(\frac{\gamma }{\beta_{0}}\right)}^{2}\right]e^{-\gamma t}.$$

Obviously, ${\tilde{E}}_{-}$ is larger than ${\tilde{E}}_{+}$, and both are decaying exponentially. We want to look at the energy difference $\Delta \tilde{E}={\tilde{E}}_{-}-{\tilde{E}}_{+}$ at the initial time t=0 and correlate it to physical properties of the environment:

(80) $$\Delta {\tilde{E}}_{0}={\tilde{E}}_{-}\left(0\right)-{\tilde{E}}_{+}\left(0\right)=\frac{\hbar }{4}\frac{\gamma^{2}}{\beta_{0}}=\frac{m}{2}\gamma^{2}\sigma_{x,0}^{2}.$$

Using again the equivalence between the diffusion coefficient (67) of our quantum mechanical description (here for t=0) and the one of the Brownian motion, the Einstein relation (68), this energy difference can be expressed as

(81) $$\Delta {\tilde{E}}_{0}=kT,$$

i.e., it is also related to the thermal energy of the environment.

For, t=0, the energy ${\tilde{E}}_{+}\left(0\right)$ is identical to ${\tilde{E}}_{L}$, the one without environment, whereas ${\tilde{E}}_{-}\left(0\right)$ obviously contains some energy transfer from the environment, similar to the situation for the damped harmonic oscillator.

### 4.2. Uncertainty Product without and with Environment

Now the uncertainty products $U=\sigma_{x}^{2}\sigma_{p}^{2}$ for the cases discussed in the previous subsection are considered using the position and momentum uncertainties determined there.

First, the harmonic oscillator without environment yields

(82) $$U_{L}=\frac{\hbar }{2m\omega_{0}}\cdot \frac{m\hbar }{2}\omega_{0}=\frac{\hbar^{2}}{4};$$

including the environment, one obtains

(83) $$U_{\mathrm{NL}}=\frac{\hbar }{2m\Omega }\cdot \frac{m\hbar }{2}\frac{\omega_{0}^{2}}{\Omega }=\frac{\hbar^{2}}{4}{\left(\frac{\omega_{0}}{\Omega }\right)}^{2}>\frac{\hbar^{2}}{4}$$

or

(84) $$U_{\mathrm{NL}}=\frac{\hbar^{2}}{4}\left[1+\frac{\gamma^{2}/4}{\Omega^{2}}\right]=\frac{\hbar^{2}}{4}\left[1+{\left(\frac{\gamma /2}{\beta_{0}}\right)}^{2}\right].$$

For the free motion, Equations (70) and (71) lead to

(85) $$U_{L}=\frac{\hbar }{2m\beta_{0}}\left[1+{\left(\beta_{0}t\right)}^{2}\right]\frac{m\hbar }{2}\beta_{0}=\frac{\hbar^{2}}{4}\left[1+{\left(\beta_{0}t\right)}^{2}\right].$$

At the initial time, $U_{L}\left(0\right)=\frac{\hbar^{2}}{4}$; i.e., the wave packet is at t=0 an MUCS. For t⟶∞, $U_{L}$ goes to infinity, $\lim_{t⟶\infty }U⟶\infty$. Including again the environment, for ${\tilde{C}}_{+}=0$ one obtains

(86) $$U_{+}=\frac{\hbar^{2}}{4}\left[1+\left\{\left(\frac{\beta_{0}}{\gamma }\right){\left(1-e^{-\gamma t}\right)}^{2}\right\}\right],$$

for ${\tilde{C}}_{-}=-\gamma$

(87) $$U_{-}=\frac{\hbar^{2}}{4}\left[1+{\left\{\left(\frac{\beta_{0}}{\gamma }\right){\left(1-e^{-\gamma t}\right)}^{2}-\left(\frac{\gamma }{\beta_{0}}\right)e^{-\gamma t}\right\}}^{2}\right].$$

For t=0, the uncertainty product becomes

(88) $$U_{-}\left(0\right)=\frac{\hbar^{2}}{4}\left[1+{\left(\frac{\beta_{0}}{\gamma }\right)}^{2}\right]>U_{+}\left(0\right)=\frac{\hbar^{2}}{4};$$

i.e., only $U_{+}\left(0\right)$ is the minimum uncertainty product; thus, the corresponding wave packet is an MUCS.

In the limit t⟶∞, both uncertainty products approach the same finite maximum value:

(89) $$U_{\pm ,\max}=U_{\pm }\left(t⟶\infty \right)=\frac{\hbar^{2}}{4}\left[1+{\left(\frac{\beta_{0}}{\gamma }\right)}^{2}\right],$$

which, for γ⟶0, goes to infinity; i.e., the result of the system without environment.

Interesting is the situation for γ⟶∞, as in this case, both maximum values approach the minimum uncertainty:

(90) $$\lim_{\gamma ⟶\infty }U_{\pm ,\max}⟶\frac{\hbar^{2}}{4}=U_{\min}=U_{L}\left(0\right).$$

The parameter γ corresponds to the frequency of collision between system and environment, i.e., the interaction of the system with the environment. This brings to mind the so-called quantum Zeno effect, a property of quantum systems that allows the slowing down of the system’s time-evolution. This causes the evolution to “freeze” in the system’s initial state by measuring it frequently. The meaning of this effect has been generalized to a suppression of the time evolution of a system not only by measurement, but also by interaction with the environment, stochastic fields, and so on. In this sense, the reduction of our uncertainty product to $\frac{\hbar^{2}}{4}$, which is the initial value of $U_{L}$ and $U_{+}$ for γ⟶∞, i.e., continuous interaction, could be interpreted as belonging to this category (for $U_{-}$, an additional initial contribution from the environment must be taken into account).

Comparison with Zeno’s paradox was first made by Misra and Sudarshan [34] in 1977, although the derivation of this effect was already presented by Degasperis et al. [35] in 1974. The question of the name being appropriate for this effect was nicely addressed by Home and Whitaker in [36]. To show the problematic aspect, we simply quote their comment: “Should the effect be called “quantum Zeno”? The original Zeno paradox was expressed in different form ([37], p. 394; [38], p. 308) both of which are based on the difficulty of building up an idea of “motion” from a series of instantaneous snapshots. The **quantum** Zeno effect is based on the idea of measurement freezing change. Thus there are rather superficial similarities but rather more deep-seated differences between the sets of ideas. More fundamentally, Newton’s second law is second order in time, while Schrödinger’s equation is first order, so there may certainly be no direct comparison of these processes. Provided this is recognised, the use of similar terminology should do no harm”.

The additional term ${\left(\frac{\beta_{0}}{\gamma }\right)}^{2}$ in the maximum value of $U_{\pm }$ can also be interpreted in another way. Taking into account that the time-dependent Schrödinger equation can be written as a diffusion equation with a purely imaginary diffusion coefficient, $D_{\mathrm{qm}}=\frac{\hbar }{2m}$, and the diffusion coefficient of our open system at initial time is given, according to (67), by $D_{0}=\frac{\gamma }{2}\sigma_{x,0}^{2}$, the additional term can be written as

(91) $${\left(\frac{\beta_{0}}{\gamma }\right)}^{2}={\left(\frac{\hbar }{\gamma 2m\sigma_{x,0}^{2}}\right)}^{2}={\left(\frac{1}{2}\frac{\frac{\hbar }{2m}}{\left(\gamma /2\right)\sigma_{x,0}^{2}}\right)}^{2}={\left(\frac{1}{2}\frac{D_{\mathrm{qm}}}{D_{0}}\right)}^{2};$$

i.e., it depends essentially on the ratio of the two diffusion coefficients.

Another interesting formal similarity emerges if the Einstein relation $D=\frac{kT}{m\gamma }$ is identified with the above-mentioned quantum diffusion coefficient $D_{\mathrm{qm}}=\frac{\hbar }{2m}$, i.e., $D=\frac{kT}{m\gamma }=\frac{\hbar }{2m}$ (which would imply $\frac{1}{2}kT=\frac{\hbar }{2}\frac{\gamma }{2}$, in agreement with Equation (69)). The application of $D=\frac{\hbar }{2m}$ as a diffusion coefficient occurs in approaches that try to explain quantum physics by classical physics. In the case of Bohmian mechanics, it was already mentioned above that the explanation in terms of classical trajectories failed. Another model is Nelson’s stochastic mechanics [39,40,41,42] based on Newtonian physics. There, a mysterious backward diffusion process, combined with the usual forward diffusion process, provides the particle’s drift—i.e., its mean position as a dynamically determined quantity, not as a classically independent variable (see also [43]). Nelson himself later on stated [42] “This makes it unrealistic to regard the trajectories as physically real.” A possible way out of this problem could be to assume “that a particle’s drift actually is a dynamically determined quantity, albeit in a new framework. This framework [⋯] would have to be some kind of steady-state, maintained by a throughput of energy from the (“contextual”) environment” [44]. In this sense, quantum mechanics is considered an emergent phenomenon.

In other words, the quantum system is embedded in some kind of environment, e.g., a random zero-point electromagnetic radiation field [45,46], a special superfluid medium [47] or a thermal environment of non-zero average temperature [44,48,49,50]. Later on, Grössing et al. adapted their model to the experiments by Couder’s group [51,52,53]. In this model, the constant energy of the quantum system necessary to remain in a non-equilibrium steady-state is supplied from the (zero-point) oscillations of the vacuum; i.e., the quantum system is assumed “to be embedded in an environment comprising a corresponding energy bath with both periodic and stochastic contributions” [50], thereby resembling a Brownian motion situation. From these assumptions, the value $D=\frac{\hbar }{2m}$ for the diffusion coefficient can be derived by comparing the diffusion constant of the sub-quantum Brownian motion with the temperature and the energy of the oscillation of the sub-quantum heat bath.

Using the same comparison between our classical Brownian motion diffusion constant and $D=\frac{\hbar }{2m}$, the imaginary part $W_{I}$ of the logarithmic nonlinearity (43) becomes

(92) $$W_{I}=-iTk\left(\ln\rho -\langle \ln\rho \rangle \right),$$

where $-k\langle \ln\rho \rangle =-k\int_{-\infty }^{+\infty }dx\ln\rho$ has a form similar to the statistical mechanics definition of entropy, S. Thus, the mean value of (quantum) mechanical energy (calculated with $\psi_{\mathrm{NL}}$), ${\langle H_{\mathrm{NL}}\rangle }_{\mathrm{NL}}={\langle T\rangle }_{\mathrm{NL}}+{\langle V\rangle }_{\mathrm{NL}}=E$, together with the second term of (43) would resemble E−iTS—i.e., similar to the expression for the free energy of thermodynamics, only here, again, with the imaginary unit i appearing in the quantum mechanical context. Since recent experiments have shown [54,55] that quantum mechanics necessarily has to be formulated in terms of complex quantities, this might not be as strange as it appears at first sight, but this aspect needs much more further investigation.

## 5. Conclusions

Using a polar ansatz for the wave function $\psi \left(x,t\right)=\sqrt{\rho }\exp\left(\frac{i}{\hbar }S\right)$ in terms of amplitude $\sqrt{\rho }$ and phase S/ℏ, Madelung [3] proposed a formulation in terms of two real equations that are formally similar to classical hydrodynamic equations, a continuity equation for the amplitude and a modified Hamilton–Jacobi equation for the phase, instead of the complex Schrödinger equation. These two equations are coupled, as the gradient of the phase occurs as a velocity (actually momentum) field in the continuity equation, and the amplitude occurs in the form of the so-called quantum potential $V_{\mathrm{qu}}$ in the modified Hamilton–Jacobi equation.

In a recent paper, we have shown how the Madelung picture can be formulated in terms of complex quantities, not only allowing for the formulation in momentum space and beyond [23], but also for its extension to other hydrodynamic properties [24]. In position space (to which the discussion in this paper is restricted), the momentum operator and its corresponding complex hydrodynamic quantity, $P=P_{R}+iP_{I}$, are of interest. The real part $P_{R}$ only depends on the phase S/ℏ; the imaginary part $P_{I}$ only on the amplitude $\sqrt{\rho }$. The situation changes when nonlinear functions of P are considered, such as for the kinetic energy, where the square of the momentum appears. The real part of the complex quantity corresponding to the kinetic energy does not only contain contributions from $P_{R}$, but also from $P_{I}$ and its derivative. Although the mean value of $P_{I}$, like all mean values of the complex Madelung quantities, vanishes, this is not the case for the contributions of $P_{I}$ to the real part of the kinetic energy.

Consequently, the uncertainty product of $\sigma_{x}^{2}$ and $\sigma_{p}^{2}$ has two contributions, one from the amplitude and one from the phase. The contribution from the amplitude provides the minimum uncertainty $\frac{\hbar^{2}}{4}$. If the uncertainty product $U=\sigma_{x}^{2}\sigma_{p}^{2}$ has a larger value, this contribution originates from the phase and can be expressed in terms of the correlation of position and momentum uncertainties via their anti-commutator.

To illustrate this situation, we use Gaussian wave packets that are exact analytic solutions of the time-dependent Schrödinger equation for the systems we consider, and that are completely determined by their maximum and width and the corresponding dynamics. The time-evolution of the maximum η(t) is determined by the classical equation of motion for the given potential; the time-evolution of the width by a complex nonlinear Riccati equation for C(t). The imaginary part of this equation determines the real part of $C_{R}$ that enters the phase of the wave function, and thus $\sigma_{p,ph}^{2}$ and $\sigma_{xp}$. The imaginary part of C(t) can be expressed in terms of a new variable α(t) that is directly proportional to the wave packet width, $C_{I}=\frac{1}{\alpha^{2}}$. By inserting $C_{I}$ and $C_{R}$ in terms of α and $\dot{\alpha }$ into the real part of the Riccati equation, the time-evolution of the wave packet width can be directly obtained from a real nonlinear equation for α(t), the so-called Ermakov equation.

Next, the influence of a dissipative environment on these systems was considered. Amongst several possible approaches to treat such open systems, we chose an effective one that is compatible with our hydrodynamic Madelung picture. Since the interaction with a dissipative environment breaks the symmetry under time-reversal of the dynamics, this should also be reflected in the corresponding equations of motion. Unlike other approaches that are based on phenomenological friction forces, like the one in the Langevin equation of Brownian motion, something that could be related to the modified Hamilton–Jacobi equation (Equation (4)), our approach starts from the continuity Equation (3), breaking the time symmetry by introducing an irreversible diffusion term, leading to a Fokker–Planck-type equation, which in position space is a Smoluchowski equation. The attempt to separate this equation for $\rho =\psi^{*}\psi$ into two equations depending only on ψ or $\psi^{*}$ is not successful in general due to the coupling of ψ and $\psi^{*}$ via the diffusion term. However, introducing an additional separation condition, where $\frac{\partial^{2}}{\partial x^{2}}\rho$ is related to $\ln\rho =\ln\psi +\ln\psi^{*}$, permits separation. Although this separation condition restricts the possible solutions of the resulting modified Schrödinger equation, it still enables Gaussian wave packets, the ones we consider in this paper, and also enables normalization of the solutions, though the diffusion term leads to a (nonlinear) imaginary term in the Hamiltonian operator, thereby changing it to a non-Hermitian one.

Due to separation, an additional real contribution is introduced into the Hamiltonian that, according to Ehrenfest, provides the desired friction force proportional to velocity or momentum, known from the Langevin equation, but, as a consequence of the separation condition, it is not introduced as a starting requirement for the construction of the additional interaction term in the Hamiltonian. Consequently, also in the second Madelung equation, the modified Hamilton–Jacobi equation, this friction force proportional to momentum appears.

As the mean value of the additional nonlinear term vanishes, 〈W〉=0, no additional observable term appears in the mean value of the Hamiltonian, and thus in the energy, i.e., $\langle H_{\mathrm{NL}}\rangle =\langle H_{L}\rangle =\langle T\rangle +\langle V\rangle$. However, these mean values are now evaluated with the solutions of the nonlinear Schrödinger equation (Equation (42)), leading to different time-dependence of the mean values of the energy and the uncertainties.

That also means that $\tilde{E}$ and U, as functions of $\sigma_{x}^{2}$, $\sigma_{p}^{2}$ and $\sigma_{xp}$, or $C_{I}$ and $C_{R}$, respectively, are unchanged, but the time dependence of $C_{I}$ and $C_{R}$ that determines that of $\sigma_{x}^{2}$, $\sigma_{p}^{2}$ and $\sigma_{xp}$, definitely depends on the additional terms from $W=W_{R}+iW_{I}$. The imaginary part $W_{I}$, originating from the Smoluchowski equation (Equation (39)) for ρ, enters the imaginary part of the Riccati equation (Equation (51)), changing $C_{R}$ according to (52). As $C_{R}$ is the quantity that enters $P_{R}$, the terms that are responsible for the addition to the minimum uncertainty product via ${\left(\sigma_{xp}\right)}^{2}$ are changed too.

The real part of the additional term, $W_{R}$, corresponding to the modified Hamilton–Jacobi equation for the phase, adds a linear term to the real part of the Riccati equation, thereby changing the Ermakov equation for the width of the wave packet and the time-dependence of the amplitude.

As examples, the harmonic oscillator and the free motion, without and with environmental interaction, have been considered. For the harmonic oscillator, the interaction with the environment provides an additional contribution to the energy that originates from the quantum uncertainties, causing it to be larger than without environment. This is similar to the situation of a Brownian particle in a bath of temperature *T*, where the final energy is $\frac{1}{2}kT$ due to the back-transfer of energy from the bath via stochastic, i.e., statistic, force, the average value of which vanishes, similarly to $\langle P_{I}\rangle =0$, the mean value of the imaginary part of our complex momentum. The mean value of $P_{I}^{2}$ does not vanish, similarly to the average value of the square of the stochastic force that supplies the final energy of the Brownian particle.

For the free motion, the interaction with the environment causes a bifurcation, so there are two states with different initial ground state energies and different uncertainty products.

The parameter that connects the quantum system via the Smoluchowski equation (Equation (39)) with the environment is the diffusion coefficient *D*. For different choices of *D*, different environments can be taken into account. By choosing a Brownian motion environment and taking into account for this case the Einstein relation $D=\frac{kT}{m\gamma }$, the excess energy of the damped harmonic oscillator and the difference between the lowest energies of the two states of the damped free motion, originating from bifurcation due to broken time-reversal symmetry, can be expressed in terms of the parameters of the environment, particularly in terms of its temperature *T*.

Taking the time-dependent Schrödinger equation for the free motion as a diffusion equation with imaginary diffusion coefficient allows formal similarity between the imaginary part of the additional nonlinear term and the definition of entropy in statistical mechanics; however, in the quantum mechanical context, the imaginary unit i appears.

In the paper concerning the uncertainties in our complex Madelung picture [12], we considered position and momentum space. Treating the corresponding open systems with our nonlinearity (43), the gradient of this term is proportional to momentum. In momentum space, however, the gradient of the logarithm of the wave function with respect to momentum would provide a friction force proportional to position, i.e., a totally different physical situation. Therefore, the treatment of open systems in momentum space needs a formally different approach that is beyond the scope of this paper but will be considered in forthcoming work.

## Data Availability

Not applicable.
